# Citric-acid dialysate improves the calcification propensity of hemodialysis patients: A multicenter prospective randomized cross-over trial

**DOI:** 10.1371/journal.pone.0225824

**Published:** 2019-12-05

**Authors:** Karlien J. ter Meulen, Marijke J. E. Dekker, Andreas Pasch, Natascha J. H. Broers, Frank M. van der Sande, Jeroen B. van der Net, Constantijn J. A. M. Konings, Isabelle M. Gsponer, Matthias D. N. Bachtler, Adelheid Gauly, Bernard Canaud, Jeroen P. Kooman

**Affiliations:** 1 Department of Internal Medicine, Division of Nephrology, Maastricht University Medical Center+, Maastricht, the Netherlands; 2 Department of Internal Medicine, Division of Nephrology, Catharina Hospital Eindhoven, Eindhoven, the Netherlands; 3 Calciscon AG, Nidau, Switzerland; 4 Fresenius Medical Care, Bad Homburg, Germany; Medizinische Universitat Graz, AUSTRIA

## Abstract

**Introduction:**

The concentration of dialysate calcium (dCa) has been suggested to affect vascular calcification, but evidence is scarce. Calcification propensity reflects the intrinsic capacity of serum to prevent calcium and phosphate to precipitate.

The use of citric-acid dialysate may have a beneficial effect on the calcification propensity due to the chelating effect on calcium and magnesium. The aim of this study was to compare the intradialytic and short-term effects of haemodialysis with either standard acetic-acid dialysate with dCa1.50 (A1.5) or dCa1.25 (A1.25), as well as citric-acid dialysate with dCa1.50 (C1.5) in bicarbonate dialysis on the calcification propensity of serum.

**Methods:**

Chronic stable hemodialysis patients were included. This multicenter randomized cross-over study consisted out of a baseline week (A1.5), followed by the randomized sequence of A1.25 or C1.5 for one week after which the alternate treatment was provided after a washout week with A1.5. Calcification propensity of serum was assessed by time-resolved nephelometry where the T_50_ reflects the transition time between formation of primary and secondary calciprotein particles.

**Results:**

Eighteen patients (median age 70 years) completed the study. Intradialytic change in T_50_ was increased with C1.5 (121 [90–152]min) compared to A1.25 (83 [43–108]min, p<0.001) and A1.5 (66 [18–102]min, p<0.001). During the treatment week, predialysis T_50_ increased significantly from the first to the third session with C1.5 (271 [234–291] to 280 [262–339]min, p = 0.002) and with A1.25 (274 [213–308] to 307 [256–337]min, p<0.001), but not with A1.5 (284 [235–346] to 300 [247–335]min, p = 0.33).

**Conclusion:**

Calcification propensity, as measured by the change in T_50_, improved significantly during treatment in C1.5 compared to A1.25 and A1.5. Long-term studies are needed to investigate the effects of different dialysate compositions concentrations on vascular calcification and bone mineral disorders.

## Introduction

Cardiovascular diseases (CVD) are still the main cause of death in hemodialysis (HD) patients despite technical advances in dialysis and better overall patient care.[1] A major component of CVD in HD patients is the presence of vascular calcifications that are independently related to all-cause and cardiovascular mortality.[1] One of the presumed mechanisms linking bone mineral disorder and vascular calcification is the formation of calciprotein particles (CPPs), and their transformation from primary to secondary CPPs. The primary CPPs are composed of fetuin-A, calcium (Ca) and phosphate as colloidal particles. These particles can spontaneously convert into hydroxyapatite-containing secondary CPPs which are suspected to cause calcification by interacting with vascular structure components.[2] The transition time (T_50_) between these particles is believed to reflect the intrinsic capacity of the serum preventing Ca and phosphate to precipitate, the so-called calcification propensity of the serum.[3] Furthermore, recent studies have shown that T_50_ is also highly predictive of all-cause mortality in patients with advanced chronic kidney disease (CKD), kidney transplant recipients and in maintenance HD patients.[4–6]

Apart from the pathophysiological processes associated with CKD, there are additional iatrogenic factors that may aggravate vascular calcification such as the use of high-dose Ca-containing phosphate binders and the prescription of higher dialysate calcium (dCa) concentrations.[7, 8] Most clinical evidence of the latter has been obtained with the comparison of dCa of 1.75mmol/l (dCa1.75) with dCa of 1.25mmol/l (dCa1.25).[9, 10] A dCa1.75 has been identified as a risk factor for all-cause cardiovascular or infection-related hospitalization.[11] This finding was confirmed in the ‘Dialysis Outcomes and Practice Patterns Study’(DOPPS) that also found an association of high dCa with all-cause mortality.[12] On the contrary, reducing dCa below 1.25mmol/l has been associated with a higher risk for hospitalization, arrhythmia and sudden cardiac death.[13, 14] The limited number of studies available, comparing dCa1.25 and 1.50mmol/l, have not yet provided conclusive results. A large French cohort showed no associations of dCa1.25, 1.50 and 1.75 with survival,[15] in contrast to He et al. who found in a small interventional study that dCa1.25 was associated with an improved survival compared to dCA1.5.[16]

Therefore, there is still equipoise about the optimal dCa in conventional dialysis with bicarbonate as main buffer to which acetic-acid dis added in (dAcet). This is reflected by the discrepancy between international guidelines as the most recent guideline of KDIGO (2017) suggests a dCa between 1.25 and 1.50.[17]

A relatively novel development in chronic HD is the use of citric-acid dialysate (dCit) in bicarbonate dialysis instead of dAcet to improve intradialytic hemodynamic stability and tolerance.[18] Citrate is also known as a chelator of Ca and magnesium (Mg).[19]

The aim of this study was to compare the intradialytic and short-term effects between acetic-acid dialysate solutions with a dCa composition of respectively 1.25mmol/l (A1.25) or 1.5mmol/l (A1.5), and a citric-acid dialysate with a dCa of 1.5mmol/l (C1.5) on the calcification propensity of serum by assessing T_50_. The hypothesis is that A1.25 is associated with an improvement in calcification tendency as compared to A1.5 and that a further improvement can be achieved by the use of dCit in bicarbonate dialysis caused by lowering the Ca overload due to the Ca chelating effect.[20]

## Materials and methods

A multicenter, randomized, cross-over trial has been conducted in two Dutch hospitals (Catharina hospital Eindhoven and Maastricht Medical University Center Maastricht). Stable HD patients were included between April and September 2017.

They were on HD for at least three months and had a stable blood access (AV-fistula/graft or central venous catheter) and a QTc-interval below 470ms recorded by a 12-lead ECG. Patients with acute ongoing illness, malignancy or uncontrolled diabetes mellitus were excluded. Written informed consent was obtained by the researchers. Baseline characteristics were attained from the medical files.

**Fig 1:**
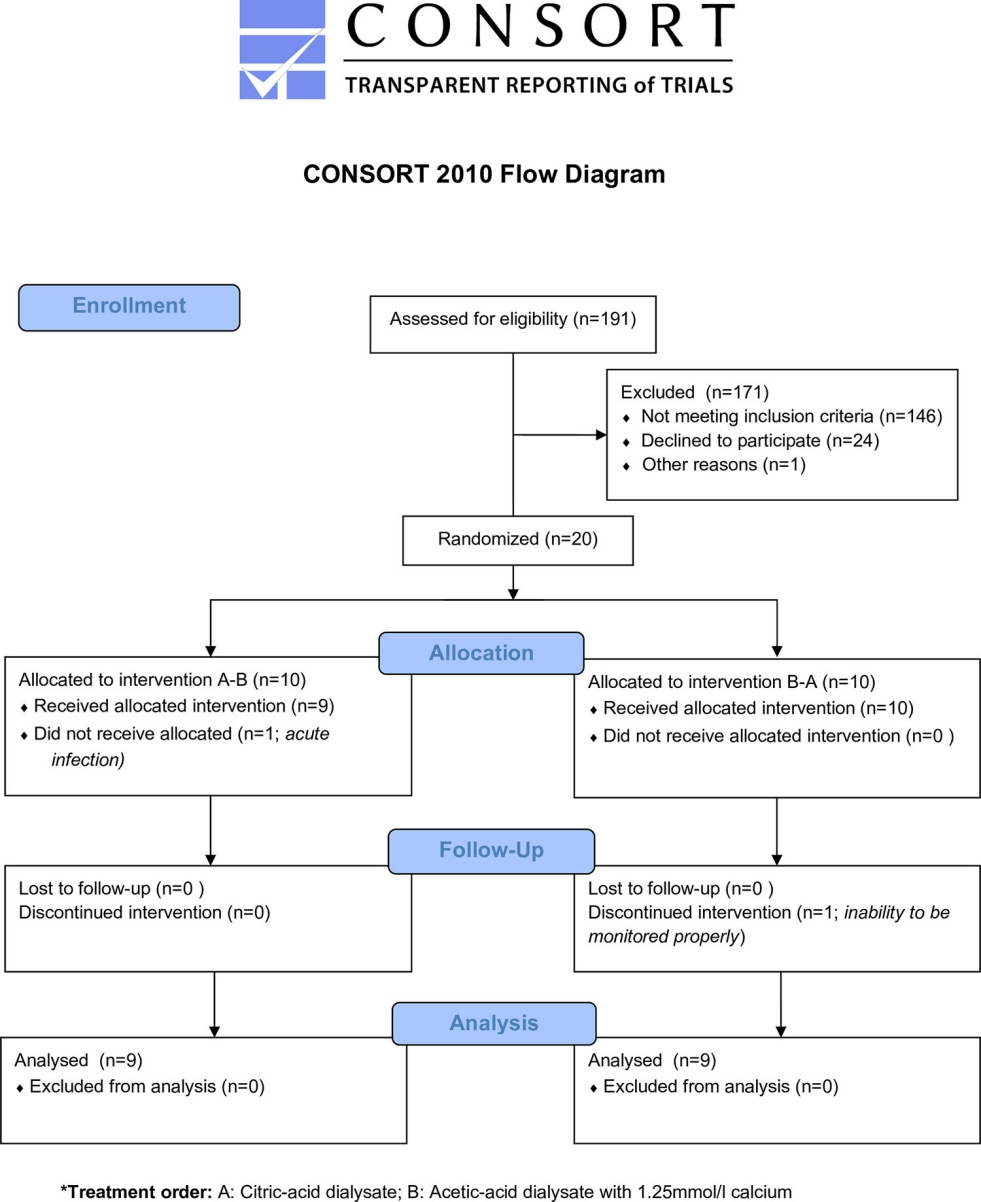
CONSORT 2010 flow diagram. Flow diagram of study. A: Citric-acid dialysate; B: Acetic-acid dialysate with 1.25mmol/l calcium.

**Fig 2:**
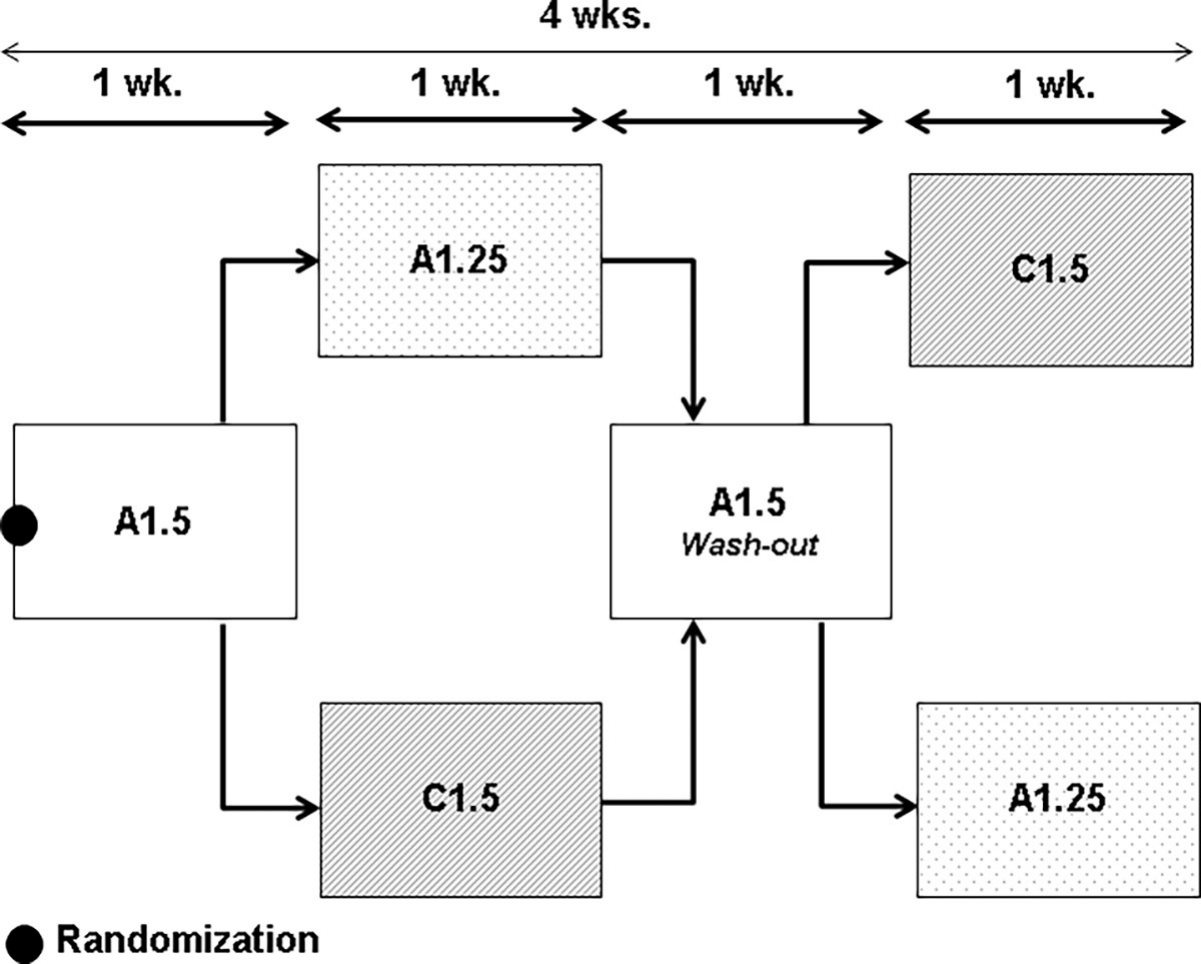
Study design. Study design per week, in total four weeks. Randomization took place before the start. A1.5 = acetic-acid dialysate with 1.50mmol/l calcium, A1.25 = acetic-acid dialysate with 1.25mmol/l calcium, C1.5 = citric-acid dialysate with 1.50mmol/l calcium.

The total duration was four weeks and consisted of a baseline week with A1.5, one week with treatment A1.25 or C1.5 (depending on randomization), one wash-out week with A1.5 and the last week with the opposite treatment that was applied in week two. The sequence was determined by online-randomization generated by the researchers. The laboratory sites were blinded for the treatment. CONSORT flow diagram is shown in Fig 1 and Study design in Fig 2.

### Dialysate composition

All treatments were bicarbonate dialyses. For all the treatments, potassium and bicarbonate were adjusted to the patient’s need. Bicarbonate was provided with a Bibag® (Fresenius) and potassium (2-3mmol/l) was modified in the dialysate. The bicarbonate concentration was kept constant for each patient throughout the study (median 32mmol/l, range 30-36mmol/l). The compositions consisted of 138.0mmol/l sodium, 0.5mmol/l Mg and 1.0g/L glucose. Ca was 1.25mmol/l in A1.25 and the other two contained 1.5mmol/l. In A1.25 and A1.5 was 3.0mmol/l acetic acid, and in C1.5 it was replaced with 1.0mmol/l citric acid.

All patients had HD thrice weekly between 3.0–4.0 hours with a minimum blood flow of 300ml/minute and a dialysate flow of 500ml/minute with a dialysis machine Fresenius 5008 Therapy System (Fresenius Medical Care, Bad Homburg, Germany). These flows were maintained during the study. All patients were using single-use high-flux membranes. Anticoagulation was applied with heparin according to the standard procedures in study centers with a reduced dose to 50% during treatment with C1.5.

### Blood sample collection and laboratory measurements

At every dialysis, blood samples were taken at the start and at the end while the patient was connected to the dialysis machine. These were analyzed for phosphate, iCa, Ca, Mg, Fetuin-A and bicarbonate, Ca concentrations were corrected for albumin according to Payne et al.[21]

The nephelometric assessments of transition from primary to secondary CPP (T_50_) were performed at Calciscon AG (Nidau, Switzerland) according to the method of Pasch et al.[3] Blood was collected in a glass vacutainer without additives and clotted for one hour at room temperature. Afterwards it was span at 2000g for 15 minutes at 20°C and aliquoted. The serum was stored and transported at 4°C until further analysis. This was a deviation of the study protocol. At the start of the actual study, the method for this measurements has been improved and therefore the serum samples did not have been frozen and been analyzed in 72 hours.

### Hemodynamic measurements

Systolic and diastolic blood pressure (SBP, DBP) was measured at the arm contralateral of the AV-fistula/graft during the dialysis sessions with an oscillometric BP monitor integrated in the dialysis machine with an interval of 30 minutes. Values were recorded during two treatments of each sequence, because detailed hemodynamic measurements were performed using a Task Force Monitor® (TFM, CN Systems, Austria) during the other treatments. Therefore it was not possible to use the BP monitor of the dialysis machine. Nadir BP was calculated as the average of the lowest values recorded per session and per patient.

### Spent dialysate

A mixture of spent dialysate and ultrafiltrate was collected continuously during each dialysis session in fractioned fashion through a connection at the outlet of the dialysis machine. At the end of each treatment, this solution was mixed and a sample of 10ml was analyzed in order to quantify solute concentration in which Ca was measured.

Ca was measured in fresh dialysate in at least three samples for each treatment. These measurements were averaged and this value was used as the fresh dialysate concentration. Measurements were taken from three consecutive dialysis.

### Solute balances

The mass balances (MB) during the dialysis were calculated with the following formula: ((D_in_ x V)–(D_out_ x V)) + (UF x D_out_), where D_in_ = concentration of solute in fresh dialysate (mmol/l), D_out_ = concentration of solute in spent dialysate (mmol/l), V = volume of dialysate (in liters; similar for fresh and spent) and UF = ultrafiltration (in liters). Volume has been calculated as: Dialysis time (minutes) x dialysate flow (ml/min). The diffusive transport has been calculated as (D_in_ x V)–(D_out_ x V). The convective transport as D_out_ x UF. Dialysis time has been rounded to thirty minute intervals. We used the average of all sessions per treatment, ranging from one to three sessions per treatment. The sessions where higher potassium was used (3.0mmol/l) where left out in this analysis due to different composition of dialysate and possible effect on transport.

### Statistical analyses

Data were expressed as median with 25^th^ and 75^th^ percentile due to the small sample size. Carryover and treatment effect were analyzed according to Wellek and Blettner.[22] Due to the small cohort, non-parametric tests were chosen. The Friedman test was applied to approach differences within and between the treatments, in case of statistical significance the Wilcoxon Signed Rank test was applied to assess the change between the sessions and between pre- and postdialysis values. Correlations between the change in laboratory values and T_50_ were tested by Spearman’s rho. All analyses were done using IBM SPSS Statistics for Windows version 23.0 (IBM Corp. Armonk, NY, USA). All tests were two-tailed and a p-value <0.05 was considered statistically significant. For the post-hoc analysis, a p-value <0.017 was considered statistically significant based on Bonferroni correction. This was based on three groups, therefore 0.05/3 = 0.017.

Median predialysis values were calculated as the average between the second and third session values per treatment. Data of the washout week was not used. Delta values were created by subtracting the predialysis of the postdialysis values per session. Delta mean values were the mean of all sessions by each treatment, except the delta for BP. In case of missing values, the mean was calculated from fewer sessions (all available data was used). Values of T_50_ and BPs were rounded to whole numbers.

The sample size was based on population of Smith et al. where T_50_ in the lowest tertile was 277±44min.[4] In a previous pilot study of our group, T_50_ in HD (n = 30) and hemodiafiltration (n = 34) were 244±64min and 253±55 min respectively.[23] By taking these populations into account, a mean of 250 min and a SD of 55 min were used for sample size calculation. With a power of 80% and the criterion for alpha set on 0.05, 19 patients would be needed to show significant differences between the different HD treatments in a pairwise analysis. A t-test was used as statistical method for calculating the target sample size. Due to the need for multiple comparisons, we originally aimed to include 22 patients in the study, we were able to include 20 patients.

This multicenter study was primarily approved by the Medical Research Ethics Committee (METC) of the Maastricht University Medical Center/Academic Hospital Maastricht (METC.151085) and secondary by the METC of Catharina hospital in Eindhoven. Both boards of directors gave approval. This study was prospectively registered in Dutch Trial Registry (NTR 5226) on the 24^th^ of April 2015. The study was monitored by Clinical Trial Center Maastricht. This study was conducted according to the principles of the declaration of Helsinki.

## Results

A total of 20 patients gave written informed consent: One patient dropped out before starting measurements due to active illness which required hospitalization. One patient was excluded during the first intervention week with A1.25 because of reaching the safety endpoint (QTc-interval >470ms) which normalized after dialysis. The data of the remaining 18 patients were included in the analyses. Table 1 shows the baseline characteristics of participants. There was no evidence for relevant carryover effects (S1 File) and therefore the cross-over design was maintained for the results.

**Table 1:** Patient and treatment characteristics.

	N = 18
Age in years	70 [57–81]
Sex, male	10 (55.6%)
Hospital, CZE	7 (38.9%)
Cause of renal failure	
Chronic renal failure, etiology unknown	2 (11.1%)
IgA-nephropathy	1 (5.6%)
Membrano-proliferative glomerulonephritis	1 (5.6%)
Congenital renal dysplasia	1 (5.6%)
Renal vascular disease	
- *due to hypertension*	3 (16.7%)
*- unspecified*	4 (22.2%)
Diabetes type 2	2 (11.1%)
Lupus erythematosus	1 (5.6%)
Other	3 (16.7%)
Dialysis vintage in months	25 [7–46]
24hr urine in ml	1292 [686–1600] (N = 13)
Kt/V, single pool	1.50 [1.40–1.89]
Ultrafiltration in ml per session	1283 [0–3139]
Smoking, yes	4 (22.2%)
Diabetes, yes	7 (38.9%)
Hypertension, yes	16 (88.2%)
Medication, yes	
Statins	12 (66.7%)
Alpha blockers	4 (22.2%)
Beta blockers	14 (77.8%)
Calcium channel blockers	9 (50.0%)
ACE inhibitor	5 (27.8%)
ARB	2 (11.1%)
Diuretics	15 (83.3%)
Nitrate	3 (16.7%)
Phosphate binders	
*- Calcium-free*	11 (61.1%)
*- Calcium containing*	1 (5.6%)
Vitamin D	16 (88.9%)
QTc-interval at start	445.5 [423–453]

Note: Data are expressed as number (percentage) or median [25^th^ -75^th^ percentile}. Frequencies are expressed in percentages. CZE: Catharina Hospital Eindhoven, ACE: angiotensin-converting-enzyme, ARB: angiotensin II receptor blocker.

### Effect of dialysate on calcification propensity measured by T_50_

**Fig 3:**
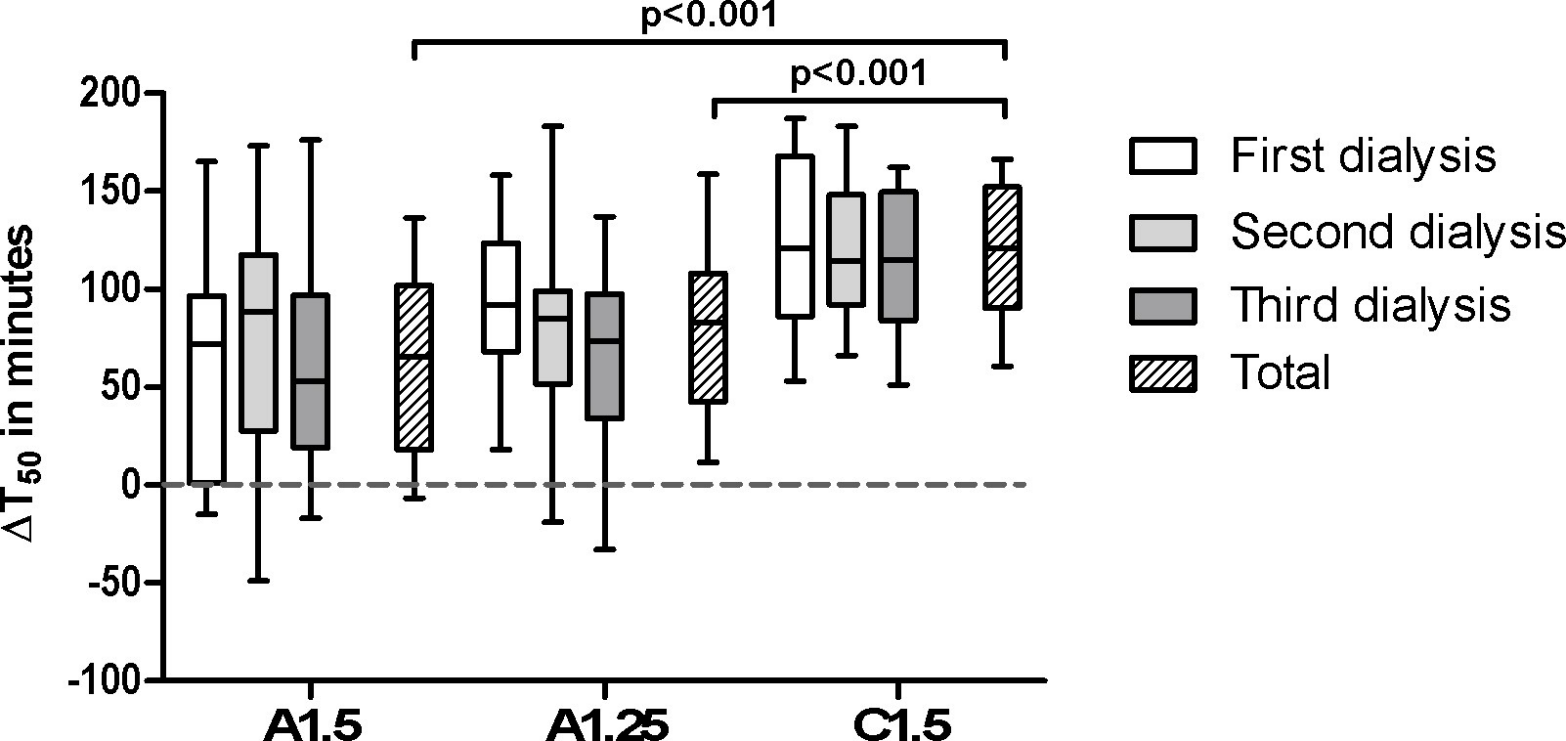
Delta transition time in minutes. Delta transition time (ΔT_50_) in minutes, displayed per dialysate and session. A1.5 = acetic-acid dialysate with 1.50mmol/l calcium, A1.25 = acetic-acid dialysate with 1.25mmol/l calcium, C1.5 = citric-acid dialysate with 1.50mmol/l calcium. P-value is between the groups.

An overview of the T_50_ is shown in Table 2. The intradialytic change (Δ) of T_50_ was significantly higher with C1.5 than with both A1.25 and A1.5 (p<0.001; Fig 3). The median postdialysis T_50_ is significantly higher for C1.5 compared to A1.25 (p<0.001) and to A1.5 (p<0.001; Table 2).

**Table 2:** Overview of transition time (T_50_) expressed in minutes.

T_50_ in minutes		A1.5	A1.25	C1.5	P-value^#^	Post-hoc (p-value, pairwise)*		
						1	2	3
**Predialysis**	*1*^*st*^	284 [235–346]	274 [213–308]	271 [234–291]				
	*2*^*nd*^	294 [230–331]	291 [239–339]]	282 [244–308]]				
	*3*^*rd*^	300 [247–335]	307 [256–337]	280 [262–339]				
	*Median*`	290 [251–323]	308 [256–337]	289 [251–320]	0.22			
	*p-value*^#^	0.33	**<0.001**	**0. 002**				
**Postdialysis**	*1*^*st*^	351 [318–399]	380 [322–400]]	408 [348–455]]				
	*2*^*nd*^	357 [337–397]	360 326–413]]	405 [352–451]				
	*3*^*rd*^	349 [316–401]	376 [327–404]]	415 [364–479]				
	*Median*	353 [323–405]	358 [327–413]	395 [368–462]	**<0.001**	0.08	**<0.001**	**<0.001**
	*p-value*^#^	0.42	0.32	0.23				
**Delta**	*1*^*st*^	72 [1–97]	92 [68–124]	121 [86–168]				
	*2*^*nd*^	89 [28–118]	75 [43–98]	115 [92–148]				
	*3*^*rd*^	53 [19–97]	74 [34–98]	115 [84–150]				
	*Median*	66 [18–102]	83 [43–108]	121 [90–152]	**<0.001**	0.09	**<0.001**	**<0.001**
	*p-value*^#^	0.34	**0.001**	0.15				

Note: Data are expressed as median with 25^th^ and 75^th^ percentile, sorted by session and in total.

`Median were calculated for predialysis from second and third session.

^*#*^P-values were calculated with Friedman test.

*Post-hoc p-values were calculated with Wilcoxon Signed Rank test as pairwise comparisons and a p-value <0.017 was considered statistically significant based on Bonferroni correction. A1.5 = acetic-acid dialysate with 1.50mmol/l calcium, A1.25 = acetic-acid dialysate with 1.25mmol/l calcium, C1.5 = citric-acid dialysate with 1.50mmol/l calcium. 1 = A1.5 vs. A1.25; 2 = A1.5 vs. C1.5; 3 = A1.25 vs. C1.5.

**Fig 4:**
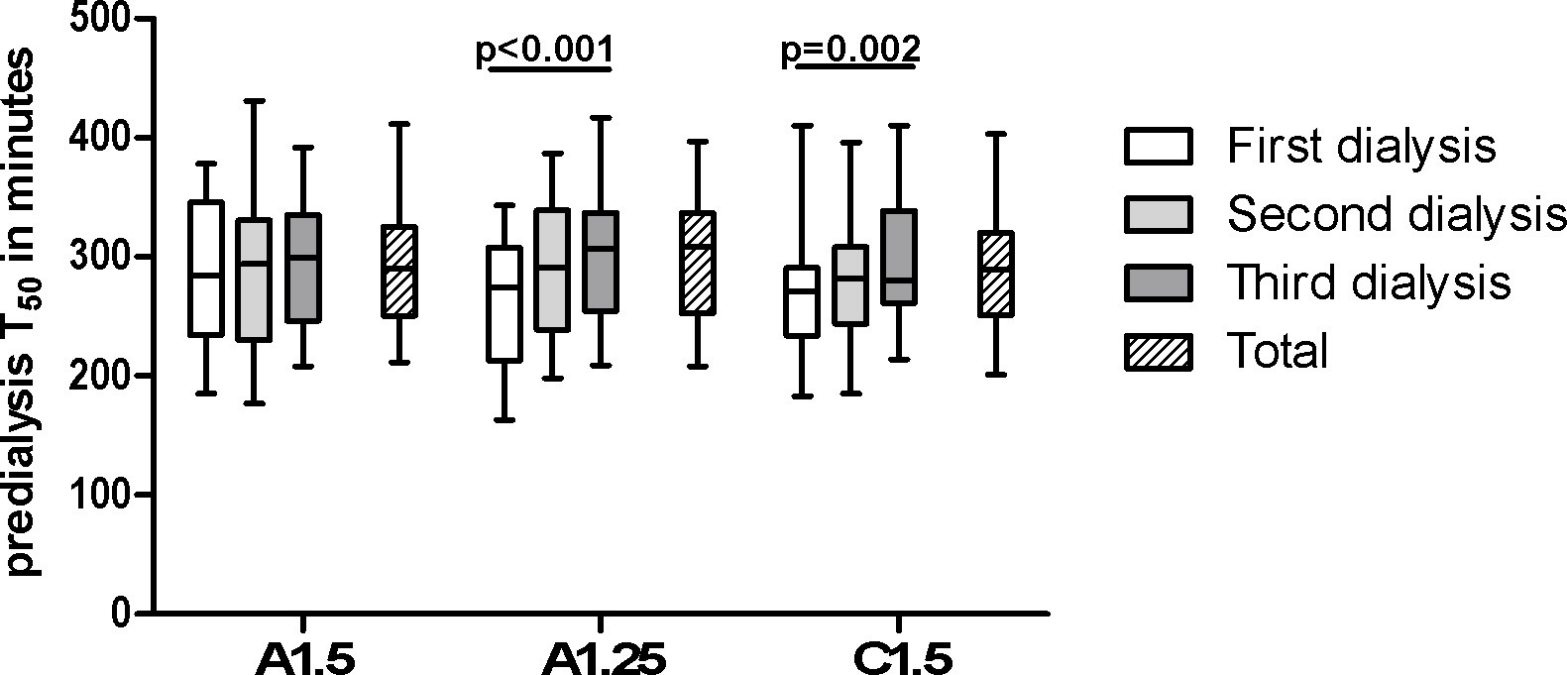
Predialysis transition time in minutes. Predialysis transition time (ΔT_50_) in minutes, displayed per dialysate and session. A1.5 = acetic-acid dialysate with 1.50mmol/l calcium, A1.25 = acetic-acid dialysate with 1.25mmol/l calcium, C1.5 = citric-acid dialysate with 1.50mmol/l calcium. P-value is shown as the trend within group.

There was a significant increase of predialysis T_50_ during the week with C1.5 (p = 0.002) and with A1.25 (p<0.001; Fig 4), but not during the week with A1.5 (p = 0.33; Table 2). There was no significant difference between median predialysis levels, expressed as the average of the second and third treatment of the week (Table 2).

### Evaluation of ΔT_50_ in correlation to laboratory values

An overview of laboratory values is given in Table 3. There was a significant smaller decrease in delta phosphate (ΔP) for A1.5 compared to A1.25 (p<0.01), and C1.5 (p = 0.005 Table 3). There was an inverse correlation noticeable between ΔT_50_ and ΔP in A1.5 (p = 0.002) and in A1.25 (p = 0.03, Table 3).

**Table 3:** Overview of laboratory values, and correlation with ΔT_50_.

Laboratory values		A1.5	A1.25	C1.5	P-value^#^	Post-hoc (p-value, pairwise)*		
						1	2	3
**Phosphate** (mmol/l)	*Predialysis*	1.38 [1.12–1.62]	1.49 [1.34–1.59]	1.47 [1.14–1.60]	0.36			
	*Postdialysis*	0.63 [0.54–0.81]	0.57 [0.53–0.066]	0.54 [0.48–0.67]	0.14			
	*Delta*	-0.84 [-0.96 - -0.64]	-0.98 [-1.05 - -0.80]	-0.94 [-1.03–0.72]	**0.04**	**<0.01**	**0.005**	0.14
	*Correlation (r; p-value)*	**-0.69; 0.002**	**-0.51; 0.03**	-0.34; 0.17				
**Ionized calcium** (mmol/l)	*Predialysis*	1.14 [1.07–1.20]	1.14 [1.10–1.20]	1.14 [1.08–1.20]	0.64			
	*Postdialysis*	1.26 [1.21–1.31]	1.13 [1.10–1.16]	1.10 [1.10–1.13]	**<0.001**	**<0.001**	**<0.001**	**<0.001**
	*Delta*	0.10 [0.07–0.14]	-0.01 [-0.05–0.01]	-0.04 [-0.06–0.02]	**<0.001**	**<0.001**	**<0.001**	0.04
	*Correlation* *(r; p-value)*	-0.15; 0.57	0.07; 0.80	-0.09; 0.75				
**Total calcium** (mmol/l)	*Predialysis*	2.30 [2.14–2.42]	2.32 [2.11–2.40]	2.32 [2.14–2.41]	0.68			
	*Postdialysis*	2.48 [2.35–2.54]	2.25 [2.21–2.32]	2.40 [2.29–2.45]	**<0.001**	**<0.001**	**<0.001**	**<0.001**
	*Delta*	0.16 [0.11–0.31]	-0.02 [-0.15–0.07]	0.08 [0.02–0.20]	**<0.001**	**<0.001**	**<0.001**	**<0.001**
	*Correlation* *(r; p-value)*	0.05; 0.86	0.18; 0.47	0.16; 0.52				
**Bicarbonate** (mmol/l)	*Predialysis*	24.3 [22.7–25.9]	25.2 [23.4–26.8]	24.3 [23.0–26.1]	0.06			
	*Postdialysis*	28.7 [27.4–30.4]	28.9 [28.0–30.8]	28.6 [27.0–30.0]	**0.01**	0.07	0.33	**0.001**
	*Delta*	5.0 [3.3–6.8]	5.3 [3.6–6.4]	4.5 [2.6–6.4]	**<0.01**	0.23	0.11	**0.007**
	*Correlation* *(r; p-value)*	-0.20; 0.44	0.02; 0.95	0.15; 0.54				

Note: Data are expressed as median with 25^th^ and 75^th^ percentile, sorted by dialysate. All predialysis values are median from second and third session. Correlation is between the delta of each laboratory value and delta T_50_, calculated with Spearman rho (showed as correlation; p-value). Total calcium is corrected for albumin by formula of Peyne.

^#^P-values were calculated with Friedman test.

*Post hoc p-values were calculated with Wilcoxon Signed Rank test as pairwise comparisons and a p-value <0.017 was considered statistically significant based on Bonferroni correction. A1.5 = acetic-acid dialysate with 1.50mmol/l calcium, A1.25 = acetic-acid dialysate with 1.25mmol/l calcium, C1.5 = citric-acid dialysate with 1.50mmol/l calcium. 1 = A1.5 vs. A1.25; 2 = A1.5 vs. C1.5; 3 = A1.25 vs. C1.5.

**Fig 5:**
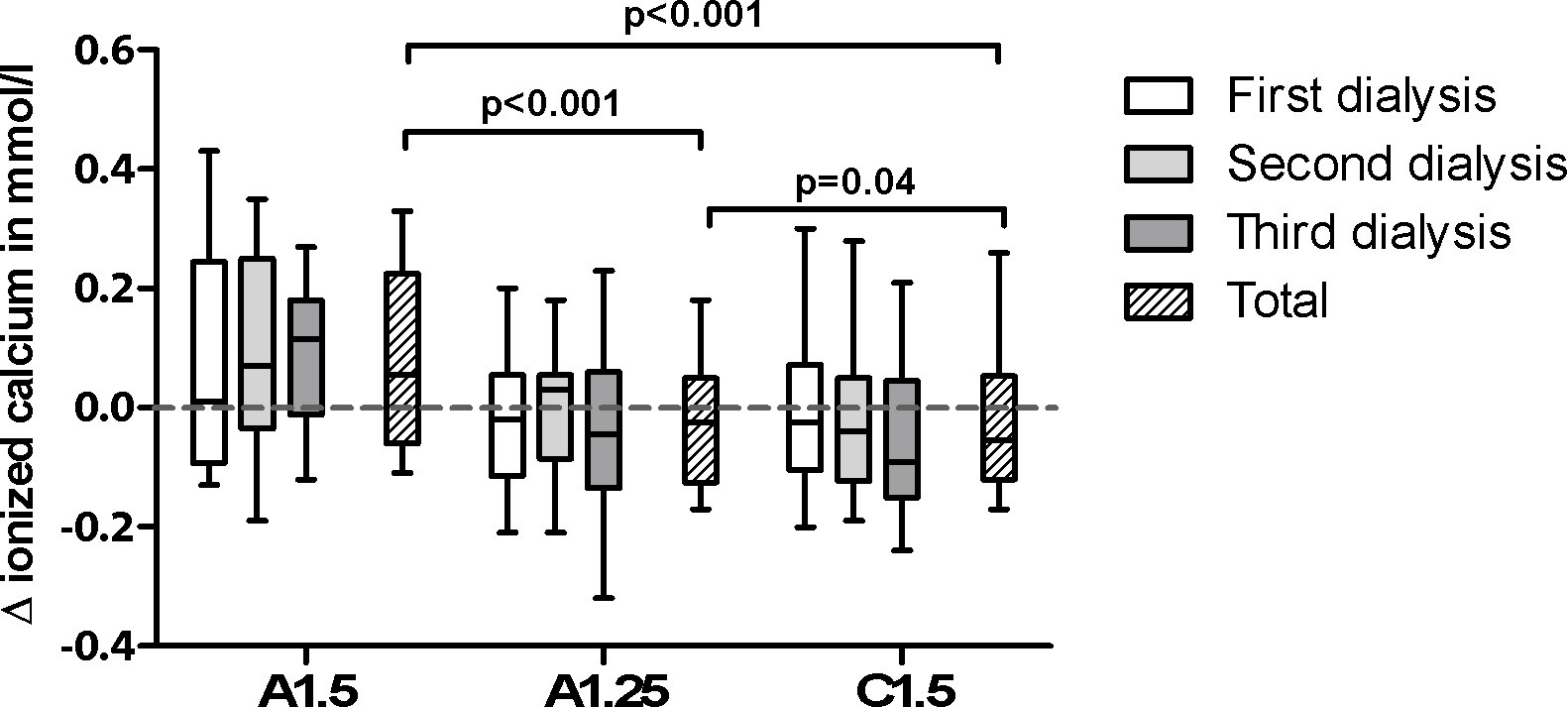
Delta ionized calcium in mmol/l. Delta ionized calcium (ΔiCa) in mmol/l, displayed per dialysate and session. A1.5 = acetic-acid dialysate with 1.50mmol/l calcium, A1.25 = acetic-acid dialysate with 1.25mmol/l calcium, C1.5 = citric-acid dialysate with 1.50mmol/l calcium. P-value is shown as the trend between the groups.

The Δionized Ca (ΔiCa) was significantly different between A1.5 and A1.25 (p<0.001), which was also observed between A1.5 and C1.5 (p<0.001) and not between A1.25 and C1.5 (p = 0.04; Table 3 and Fig 5). There was no significant correlation between ΔT_50_ and ΔiCa with all study dialysates (Table 3).

There was a significant decrease in postdialysis total Ca between A1.25 and A1.5 (p<0.001; Table 3), this was also observed between A1.25 and C1.5 (p<0.001; Table 3). The other dialysates showed both a significant increase (Table 3). The same accounts for the delta total Ca (p<0.001; Table 3).

The Δbicarbonate is significant raised in A1.25 compared to C1.5 (p = 0.007, Table 3). Postdialysis bicarbonate was significantly higher in A1.25 compared to C1.5 (p = 0.001, Table 3).

### Hemodynamic parameters

SBP, DBP and heart rate (HR) are shown in Table 4. Postdialysis SBP was significantly lower in A1.25 compared to A1.5 (p = 0.004; Table 4). There was a significant lowering of the nadir SBP with A1.25 (p = 0.004) compared to A1.5 (Table 4). Regarding the nadir DBP there was a significant decrease for C1.5 compared to A1.5 (p = 0.02; Table 4). There were no significant differences in the predialysis hemodynamic parameters.

**Table 4:** Overview of hemodynamic parameters.

Hemodynamic parameters		A1.5	A1.25	C1.5	P-value^#^	Post-hoc (p-value, pairwise)*		
						1	2	3
**Systolic BP** (mmHg)	*Predialysis*	132 [123–143]	132 [119–142]	135 [118–145]	0.22			
	*Postdialysis*	144 [115–159]	135 [117–141]	134 [119–141]	**<0.01**	**0.004**	0.04	0.70
	*Delta*	13[-7–20]	5[-11–15]	3[-18–14]	0.21			
	*Nadir*	117 [101–127]	105 [96–116]	105 [94–122]	**<0.01**	**0.004**	0.03	0.97
**Diastolic BP** (mmHg)	*Predialysis*	61 [55–71]	62 [50–69]	63 [50–70]	0.70			
	*Postdialysis*	64 [55–71]	59 [49–71]	60 [54–68]	0.36			
	*Delta*	1.5 [-5–9]	0.8 [-7–6]	4 [-5–7]	0.58			
	*Nadir*	51 [44–57]	47 [43–51]	46 [39–54]	**0.02**	0.06	0.02	0.28
**Heart rate** (/minute)	*Predialysis*	70 [63–77]	68 [58–78]	67 [62–73]	0.40			
	*Postdialysis*	67 [58–79]	69 [60–79]	69 [64–75]	0.68			
	*Delta*	-2 [7–5]	4 [1–7]	1 [-3–6]	0.09			
	*Nadir*	61 [50–66]	59 [51–69]	63 [54–66]	0.74			

Note: Data are expressed as median with 25^th^ and 75^th^ percentile.

^#^P-values were calculated with Friedman test.

*Post hoc p-values were calculated with Wilcoxon Signed Rank test as pairwise comparisons and a p-value <0.017 was considered statistically significant based on Bonferroni correction. A1.5 = acetic-acid dialysate with 1.50mmol/l calcium, A1.25 = acetic-acid dialysate with 1.25mmol/l calcium, C1.5 = citric-acid dialysate with 1.50mmol/l calcium. 1 = A1.5 vs. A1.25; 2 = A1.5 vs. C1.5; 3 = A1.25 vs. C1.5.

### Calcium mass balances

There was a positive CaMB in A1.5 that was significant different as compared with A1.25 (p<0.001), and also with C1.5 (p<0.001; Table 5). The other dialysates had a negative CaMB. This was similar for the diffusive transport with a significant difference between A1.5 and A1.25 (p<0.001), and also between A1.5 and C1.5 (p<0.001; Table 5). For the convective transport, there is a negative CaMB for all dialysates with a significant difference between A1.5 and A1.25 (p = 0.03), and none with C1.5.

**Table 5:** Calcium mass balances.

		A1.5	A1.25	C1.5	P-value^#^	Post-hoc (p-value, pairwise)*		
						1	2	3
**Calcium** mmol/treatment	*Total*	5.67 [0.59; 9.54]	-2.4 [-6.19; 1.78]	-2.00 [-5.25; -0.18]	**<0.001**	**<0.001**	**<0.001**	0.91
	*Diffusive*	6.4 [3.1; 10.2]	-1.80 [-5.8; 3.4]	-1.60 [-3.6; 1.8]	**<0.001**	**<0.001**	**<0.001**	0.88
	*Convective*^	-3.45 [-4.165; -1.62]	- 2.84 [-3.33; -1.25]	-2.90 [- 3.64; -1.42]	**<0.01**	0.03	0.11	0.18
	*Correlation (r; p-value)*	-0.08; 0.74	-0.15; 0.95	-0.03; 0.91				

Note: Data are expressed as median with 25^th^ and 75^th^ percentile.

^Patients without ultrafiltration during dialysis were left out.

^#^P-values were calculated with Friedman test.

*Post hoc p-values were calculated with Wilcoxon Signed Rank test as pairwise comparisons and a p-value <0.017 was considered statistically significant based on Bonferroni correction. Correlation is between the delta calcium mass balance and delta T_50_, calculated with Spearman rho (showed as correlation; p-value). A1.5 = acetic-acid dialysate with 1.50mmol/l calcium, A1.25 = acetic-acid dialysate with 1.25mmol/l calcium, C1.5 = citric-acid dialysate with 1.50mmol/l calcium. 1 = A1.5 vs. A1.25; 2 = A1.5 vs. C1.5; 3 = A1.25 vs. C1.5.

### Additional analysis

The ΔMg was significantly higher for C1.5 compared to A1.5 (p = 0.005) and to A1.25 (p = 0.02; S1 Table). Predialysis Mg was significant different between C1.5 and A1.5 (p = 0.04) and A1.25 (p = 0.001; S1 Table). There was no significant difference in Δfetuin-A (S1 Table).

Patients with a predialysis phosphate value below 0.70mmol/l received phosphate supplementation in dialysate during dialysis. The influence of phosphate administration was investigated and showed a slight increase of ΔT_50_ and ΔP (S2 and S3 Tables). Nonetheless, this did not affect the major outcomes of the study.

S1 File shows that there is no carryover effect.

## Discussion

In this randomized cross-over study, we found that C1.5 has a positive effect on the calcification propensity during dialysis treatment as compared to conventional dialysate solutions A1.5 and A1.25. Changes in phosphate during the different treatments were also significantly inversely related to ΔT_50_ during the dialysis treatment. To our knowledge, this is the first cross-over study to investigate the effect of different dialysis solutions on calcification propensity.

Whereas ΔT_50_ was significantly higher during C1.5 compared to A1.5 and A1.25, this was not in during A1.25 as compared to A1.5. In addition, we did not observed a significant correlation regarding ΔiCa and ΔT_50_ in all study dialysates. It is important to note that the change in ionized Ca appeared almost similar between C1.5 and A1.25, even though the dCa of C1.5 was 1.50mmol/l. Previous studies also showed a decrease of ionized Ca with the use of C1.5 due to its chelating effect on ionized Ca and Mg.[24, 25]

On the other hand, there appeared to be a persistent effect of C1.5 and A1.25 on calcification propensity persisted beyond the dialysis treatment itself, as demonstrated by an increase in predialysis ΔT_50_ during the week in which C1.5 and A1.25 were administrated as compared to the treatment with A1.5. Given the fact that the effects of C1.5 likely subsides after the end of dialysis, we suggest that the positive effects of A1.25 and C1.5 on predialysis T_50_ are most likely related to differences in Ca mass balance as compared to A1.5, whereas the effect on ΔT_50_ during the dialysis treatment is related to the additional effects of citrate on calcification propensity which extend beyond its effects on Ca mass balance.

Our results are in agreement with those of Lorenz et al., who observed an increase in T_50_ after switching from acetic-acid to citric-acid containing solutions during a three month follow up period.[20] The design of both studies differs significantly in the sense that our study was a short term randomized cross-over trial including a comparison between citric-acid with two acetic-acid containing solutions whereas the study of Lorenz et al. compared two solutions in a pre-post quasi interventional design. Therefore, both studies add significant and non-overlapping information on this topic.

As shown in our previous study,[23] changes in serum phosphate during the respective treatment were inversely related to ΔT_50_ during dialysis, which is next to its prognostic value an additional argument for the biological plausibility of this method. There was an increased phosphate removal for C1.5 as compared to A1.5, which might be caused by improved solute removal, as seen in Kossmann et al. with decline in predialysis concentrations of urea, phosphate and creatinine,[26] that can contribute to improvement of T_50_. This was also observed in Schmitz et al.[18]

Unexpectedly, we did not observe any correlation between ΔT_50_ and ΔMg, which is in contrast to the positive correlation observed in the study of Dekker et al.[23] It would be expected that due to anti-calcifying effect of Mg, an increase in serum Mg levels should cause delay of crystallization (i.e. increased T_50_).[3] However, in the present study dialysate Mg levels of 0.5mmol/l were used which led to a general decline in serum Mg levels during dialysis as also observed in earlier studies.[18] Therefore, we suggest that this information should be interpreted with great caution, certainly in view of the recent data of Bressendorf et al. who observed an improvement in calcification propensity after switching DMg from 0.5 to 1.0mmol/l.[19]

Gabutti et al. showed that an increase of bicarbonate is associated with citrate dialysate.[24] This can also affect the calcium kinetics as calcium binding on albumin is related to pH and bicarbonate concentration, and therefore there might be a change of the equilibrium of calcium during dialysis (ionized, protein-bound and bone-sequestered).[27, 28] Besides this, it has also been shown that changes in calcium levels during dialysis are dependent on a rapidly exchangeable calcium pool, which was not assessed in the present study.[29] We did not find a significant correlation between CaMB and T_50_. However we suggest that the difference in CaMB between the dialysates might be due to the fact that iCa, driving force for diffusion, is altered by citrate component.

The prescription of the dialysate appeared to affect the hemodynamic response during HD as shown in previous studies.[24, 30] In this respect, the changes in the SBP during dialysis were higher during A1.5 and A1.25 as compared to C1.5, whereas the nadir SBP was lower in C1.5 and A1.25 compared to A1.5. These were not significantly different when taking a correction for multiple comparisons into account in the post-hoc test. Further studies in hypotensive-prone HD patients would be needed to assess the relevance of these findings in more susceptible patients. Gründstorm et al. found similar results in their short-term randomized cross-over trial (n = 20) which showed after one hour in treatment a significant lower mean arterial pressure for C1.5 compared to A1.5.[25] Another study showed a higher decrease in SBP (14±4mmHg vs. 8±2mmHg; p = 0.042) and frequency of cramps (8.4% of the HD sessions vs. 1%; p<0.001) during citric-acid dialysate with dCa of 1.25mmol/l compared to A1.25.[31] Therefore, we suggest that the dialysate prescription should always be individualized by balancing the advantages and potential side effects of different treatment strategies including oral divalent ions intake (supplement or phosphate binders).

There are limitations of the present study. Primarily, it was a short-term study, assessing the effects of the different dialysate compositions during one week of treatment. The blood analyses only took place before and after dialysis, therefore the possible rebound effect of Ca, that can occur up to 180 minutes postdialysis, was not taken into account.[32] The strength of our study was the use of a cross-over design; therefore we eliminated patient variation as they served as their own control. The washout-period was long enough as no carryover effect was found. Parathyroid hormone (PTH) and inflammation markers were not measured in our study which could have an effect on the T_50_ as citrate has been shown to decrease inflammation.[33]

In conclusion, C1.5 improves calcification propensity, as measured by the change in T_50_, compared to A1.5 and A1.25 with effects lasting beyond dialysis treatment. Changes in ionized Ca and phosphate also affect calcification propensity during HD. Long-term studies with larger sample sizes are needed to investigate whether this effect will continue if the intervention period is extended.

## Supporting information

S1 Table Additional laboratory values. Data are expressed as median with 25^th^ and 75^th^ percentile. Predialysis values are median from second and third session. ^#^P-values were calculated with Friedman test. ˚Post-hoc p-values were calculated with Wilcoxon Signed Rank test. A1.5 = acetic-acid dialysate with 1.50mmol/l calcium, A1.25 = acetic-acid dialysate with 1.25mmol/l calcium, C1.5 = citric-acid dialysate with 1.50mmol/l calcium. 1 = A1.5 vs. A1.25; 2 = A1.5 vs. C1.5; 3 = A1.25 vs. C1.5.
(PDF)

S2 Table Overview of transition time (T_50_), excluding sessions with phosphate administration. A1.5 = acetic-acid dialysate with 1.50mmol/l calcium, A1.25 = acetic-acid dialysate with 1.25mmol/l calcium, C1.5 = citric-acid dialysate with 1.50mmol/l calcium. Sessions in which patients received phosphate administration (n = 2) were handled as missing data and therefore left out the analysis to see the influence phosphate administration on T_50_. Data are expressed as median with 25^th^ and 75^th^ percentile, sorted by session and in total. `Median was calculated for predialysis from second and third session. P-values were measured with Friedman test. ˚Post hoc p-values were calculated with Wilcoxon Signed Rank test (1 = A1.5 vs. A1.25; 2 = A1.5 vs. C1.5; 3 = A1.25 vs. C1.5).
(PDF)

S3 Table Overview of phosphate, excluding sessions with phosphate administration. A1.5 = acetic-acid dialysate with 1.50mmol/l calcium, A1.25 = acetic-acid dialysate with 1.25mmol/l calcium, C1.5 = citric-acid dialysate with 1.50mmol/l calcium. Data are expressed as median with 25^th^ and 75^th^ percentile in total. Correlation is between the delta value and delta T_50_, calculated with Spearman rho (showed as correlation; p-value). *P-values were measured with Friedman test. ˚Post hoc p-values were calculated with Wilcoxon Signed Rank test (1 = A1.5 vs. A1.25; 2 = A1.5 vs. C1.5; 3 = A1.25 vs. C1.5.
(PDF)

S1 File Analysis of carryover and treatment effect. (PDF)

S2 File CONSORT 2010 checklist. (PDF)

S3 File Study protocol. (PDF)
