# Supplementary material for: Citric-acid dialysate improves the calcification propensity of hemodialysis patients: A multicenter prospective randomized cross-over trial
Source: PLoS One. 2019 Dec 5;14(12):e0225824. doi: 10.1371/journal.pone.0225824 (PMC6894765; doi:10.1371/journal.pone.0225824)
Supplement: S1 Table — Data are expressed as median with 25th and 75th percentile. Predialysis values are median from second and third session. #P-values were calculated with Friedman test. ˚Post-hoc p-values were calculated with Wilcoxon Signed Rank test. A1.5 = acetic-acid dialysate with 1.50mmol/l calcium, A1.25 = acetic-acid dialysate with 1.25mmol/l calcium, C1.5 = citric-acid dialysate with 1.50mmol/l calcium. 1 = A1.5 vs. A1.25; 2 = A1.5 vs. C1.5; 3 = A1.25 vs. C1.5. (PDF) [file pone.0225824.s001.pdf]

Laboratory values		A-Ca1.50	A-Ca1.25	C-Ca1.50	P-value#	**Post-hoc** (p-value)*		
						**1**	**2**	**3**
**Magnesium** (mmol/l)	*Predialysis*	0.90 [0.83 – 0.95]	0.90 [0.82 – 0.97]	0.86 [0.79 – 0.91]	**0.001**	0.27	**0.04**	**0.001**
	Postdialysis	0.79 [0.72 – 0.81]	0.78 [0.77 – 0.80]	0.71 [0.70 – 0.74]	**<0.001**	0.54	**<0.001**	**<0.001**
	Delta	-0.12 [-0.20 - -0.07]	-0.13 [-0.19 - -0.08]	-0.15 [-0.21 - -0.11]	**0.005**	0.65	**0.005**	**0.02**
	Correlation (r; p-value)	0.13; 0.62	0.03; 0.92	-0.05; 0.86				
**Fetuin-A** (mg/ml)	*Predialysis*	0.39 [0.36 – 0.44]	0.38 [0.34 – 0.42]	0.40 [0.37 – 0.41]	0.40			
	Postdialysis	0.42 [0.38 – 0.44]	0.40 [0.37 – 0.42]	0.41 [0.39 – 0.43]	0.68			
