# Supplementary material for: Citric-acid dialysate improves the calcification propensity of hemodialysis patients: A multicenter prospective randomized cross-over trial
Source: PLoS One. 2019 Dec 5;14(12):e0225824. doi: 10.1371/journal.pone.0225824 (PMC6894765; doi:10.1371/journal.pone.0225824)
Supplement: S2 Table — A1.5 = acetic-acid dialysate with 1.50mmol/l calcium, A1.25 = acetic-acid dialysate with 1.25mmol/l calcium, C1.5 = citric-acid dialysate with 1.50mmol/l calcium. Sessions in which patients received phosphate administration (n = 2) were handled as missing data and therefore left out the analysis to see the influence phosphate administration on T50. Data are expressed as median with 25th and 75th percentile, sorted by session and in total. `Median was calculated for predialysis from second and third session. P-values were measured with Friedman test. ˚Post hoc p-values were calculated with Wilcoxon Signed Rank test (1 = A1.5 vs. A1.25; 2 = A1.5 vs. C1.5; 3 = A1.25 vs. C1.5). (PDF) [file pone.0225824.s002.pdf]

T_{50} in minutes		**A-Ca1.50**	**A-Ca1.25**	**C-Ca1.50**	**P-value**#	**Post-hoc** (p-value)*****		
						**1**	**2**	**3**
**Predialysis**	*1st*	284 [235-346]	274 [213-308]	271 [234-291]				
	2nd	290 [225-328]	276 [229-333]	282 [244-308]				
	3rd	292 [243-338]	305 [252-338]	288 [258-344]				
	Median`	290 [251-326]	308 [243-337]	289 [251-320]	0.47			
	p-value#	0.33	**<0.001**	**0.004**				
**Postdialysis**	*1st*	349 [316-396]	280 [322-400]	408 [348-455]				
	2nd	355 [333-400]	365 [327-417]	405 [352-451]				
	3rd	352 [322-407]	381 [331-407]	423 [376-485]				
	Median	353 [323-412]	371[327-413]	396 [368-462]	**<0.001**	0.13	**<0.001**	**0.001**
	p-value#	0.10	**0.01**	0.27				
**Delta**	*1st*	72 {1-97]	92 [68-124]	121 [86-168]				
	2nd	94 [35-122]	85 [52-99]	115 [92-148]				
	3rd	53 [19-97]	75 [39-99]	117 [86-105]				
	Median	66 [34-102]	87 [53-108]	121 [91-152]	**<0.001**	0.23	**<0.001**	**<0.001**
	p-value#	0.33	**0.01**	**0.02**				
