# Supplementary material for: Citric-acid dialysate improves the calcification propensity of hemodialysis patients: A multicenter prospective randomized cross-over trial
Source: PLoS One. 2019 Dec 5;14(12):e0225824. doi: 10.1371/journal.pone.0225824 (PMC6894765; doi:10.1371/journal.pone.0225824)
Supplement: S3 Table — A1.5 = acetic-acid dialysate with 1.50mmol/l calcium, A1.25 = acetic-acid dialysate with 1.25mmol/l calcium, C1.5 = citric-acid dialysate with 1.50mmol/l calcium. Data are expressed as median with 25th and 75th percentile in total. Correlation is between the delta value and delta T50, calculated with Spearman rho (showed as correlation; p-value). *P-values were measured with Friedman test. ˚Post hoc p-values were calculated with Wilcoxon Signed Rank test (1 = A1.5 vs. A1.25; 2 = A1.5 vs. C1.5; 3 = A1.25 vs. C1.5. (PDF) [file pone.0225824.s003.pdf]

Laboratory values		A-Ca1.50	A-Ca1.25	C-Ca1.50	P-value[#]	Post-hoc (p-value)*		
						**1**	**2**	**3**
**Phosphate** (mmol/l)	*Predialysis*	1.38 [1.12-1.62]	1.51 [1.39-1.59]	1.47 [1.14-1.60]	0.19			
	Postdialysis	0.63 [0.54-0.75]	0.57 [0.52-0.67]	0.54 [0.45-0.67]	0.23			
	Delta	-0.84 [-0.96 - -0.64]	-0.98 [-1.05 - -0.80]	-0.94 [-1.03 – -0.72]	**0.02**	**0.003**	**0.005**	0.08
	Correlation	**-0.67; 0.002**	-0.37; 0.14	-0.32; 0.19				
