# Supplementary material for: Citric-acid dialysate improves the calcification propensity of hemodialysis patients: A multicenter prospective randomized cross-over trial
Source: PLoS One. 2019 Dec 5;14(12):e0225824. doi: 10.1371/journal.pone.0225824 (PMC6894765; doi:10.1371/journal.pone.0225824)
Supplement: S1 File — (PDF) [file pone.0225824.s004.pdf]

Supplemental File 1: Analysis of carryover and treatment effect

Due to the use of cross-over design, it is important to evaluate if there is a carryover or treatment effect. These analyses were based on the article of Wellek and Blettner[1].*

- Test to check for assumption carryover effects

  Within-subject sums (C) is calculated as a sum of "delta T_{50} (period 1) + delta T_{50} (period 2)". Unpaired t-test was applied and showed $p=0.79$.

- Test for differences between treatment differences:

  Within-subject differences(D) are determined as "delta T_{50} (period 1) - delta T_{50} (period 2)". Unpaired t-test was used and had $p<0.001$.

With these analyses, we can conclude that there is significant improvement of dCitCa1.50 compared to dAcetCa1.25; no evidence of relevant carryover effects.

Supplemental Table 1: Overview of delta transition time (ΔT_{50}) in minutes, sorted by sequence

		A-Ca1.25 - C-Ca1.50 (N=9)	C-Ca1.50-A-Ca1.25 (N=9)
ΔT_{50}(min)	A-Ca1.50	71±45	55±44
	A-Ca1.25	79±38	75±46
	C-Ca1.50	123±39	117±33

Note: Data are expressed as mean±SD. A-Ca1.25 = acetic-acid dialysate with calcium concentration (dCa) 1.25mmol/l; A-Ca1.50 = acetic-acid dialysate with dCa 1.50mmol/l; C-Ca1.50 = citric-acid dialysate with dCa 1.50mmol/l.

[1] Wellek, S, Blettner, M: On the proper use of the crossover design in clinical trials: part 18 of a series on evaluation of scientific publications. *Dtsch Arztebl Int,* 109**:** 276-281, 2012.
