# Supplementary material for: Citric-acid dialysate improves the calcification propensity of hemodialysis patients: A multicenter prospective randomized cross-over trial
Source: PLoS One. 2019 Dec 5;14(12):e0225824. doi: 10.1371/journal.pone.0225824 (PMC6894765; doi:10.1371/journal.pone.0225824)
Supplement: S3 File — (PDF) [file pone.0225824.s006.pdf]

Effect of calcium and citrate dialysate concentrations on the calcification propensity in hemodialysis; a prospective randomized controlled cross-over trial.

Marijke JE Dekker MD[1,2], Frank M van der Sande MD PhD[1], Prof. Jeroen P Kooman MD PhD[1], Constantijn JAM Konings MD PhD[2], Andreas Pasch MD PhD[3], Prof. Karel ML Leunissen MD PhD[1], Prof. Bernard Canaud MD PhD[4].

[1] Department of Internal Medicine, Division of Nephrology, Maastricht University Medical Center, Maastricht, The Netherlands. [2] Department of Internal Medicine, Division of Nephrology, Catharina Hospital Eindhoven, Eindhoven, The Netherlands. [3] University Hospital Bern, Bern, Switzerland. [4] Fresenius Medical Care, Bad Homburg, Germany.

Correspondence to:

Marijke JE Dekker MD,

Department of Internal Medicine, Division of Nephrology Maastricht University Medical Center,

Maastricht University Medical Center,

P. Debeyelaan 25

PO BOX 5800

6202 AZ Maastricht, The Netherlands

Protocol ID	
Short title	The calcification propensity of uremic patients on hemodialysis.
Version	5.0
Date	27-06-2017
Coordinating investigator/project leader	M.J.E. Dekker MD
Principal investigator(s)	M.J.E. Dekker MD, Prof. J.P. Kooman MD PhD, F.M. van der Sande MD PhD.<br><br>Division of Nephrology, Department of Internal Medicine<br>Maastricht University Medical Center PO<br>Box 5800<br>6202 AZ Maastricht, The Netherlands<br><br>C.J.A.M. Konings MD PhD.<br>Division of Nephrology, Department of Internal Medicine<br>Catharina Hospital Eindhoven<br>5623 EJ Eindhoven, The Netherlands
Sponsor	Academisch Ziekenhuis Maastricht<br>PO Box 5800<br>6202 AZ Maastricht, The Netherlands
Independent physician(s)	J. Tordoir MD PhD<br>Department of Surgery<br>Maastricht University Medical Center, The Netherlands<br><br>L. Gilissen MD PhD<br>Department of Gastroenterology<br>Catharina Hospital Eindhoven, The Netherlands
Laboratory sites	Maastricht University Medical Center, Maastricht, The Netherlands

	Catharina Hospital Eindhoven, Eindhoven, The Netherlands<br><br>Andreas Pasch MD PhD<br>University Hospital Bern, Bern, Switzerland
Pharmacy	

Protocol signature sheet

Name	Signature	Date
Sponsor or legal representative<br><br>Head of Department<br>Prof. C.D.A. Stehouwer MD<br>PhD azM		
Coordinating Investigator/Project leader/Principal Investigator<br>MJE Dekker MD azM		

Table of contents

Summary p. 7

1. Introduction and rationale p. 9
2. Objectives p. 12
3. Study Design p. 12
4. Study Population p. 13
   - 4.1 Population
   - 4.2 Inclusion criteria
   - 4.3 Exclusion criteria
   - 4.4 Sample size calculation
5. Non-Investigational Product p. 14
6. Methods p. 14
   - 6.1 Study parameters/endpoints
     - 6.1.1 Primary endpoint
     - 6.1.2 Safety endpoints
   - 6.2 Randomisation
   - 6.3 Study procedures
   - 6.4 Withdrawal of individual subjects
   - 6.5 Replacement of individual subjects after withdrawal
   - 6.6 Follow-up of subjects withdrawn from treatment
   - 6.7 Premature termination of the study
7. Safety reporting p. 17
8. Statistical analysis p. 19
9. Ethical considerations p. 19
10. Administrative aspects and publication p. 21

References p. 22

List of abbreviations and relevant definitions

AE	Adverse Event
AF	Alkaline phosphate
AV-fistula	arteriovenous fistula
Ca	Calcium
Cbfa1	core binding factor alpha-1
CHD	conventional high flux
CKD	chronic kidney disease
CCMO	Central Committee on Research Involving Human Subjects<br>(in Dutch: Centrale Commissie Mensgebonden Onderzoek)
CPPs	calciprotein particles
CVVHDF	continuous veno-venous hemodiafiltration
DCa	dialysate calcium
DSMB	Data Safety Monitoring Board
FGF-23	Fibroblast growth factor 23
ESRD	end-stage renal disease
HD	hemodialysis
HDF	hemodiafiltration
HF	hemofiltration
HRV	heart rate variability
METC	Medical Research Ethics Committee (MREC)<br>(in Dutch: Medisch Ethische Toetsing Commissie (METC))
MW	molecular weight

IMP	Investigational Medicinal Product
OL-HDF	online hemodiafiltration
P	phosphate
PTH	parathyroid hormone
PWV	pulse wave velocity
SAE	serious adverse event
SUSAR	suspected unexpected serious adverse reaction
STS	sodium thiosulfate ($Na_2S_2O_3$)
T50	transition time
WMO	Medical Research Involving Human Subjects Act (in Dutch: Wet Medisch Wetenschappelijk Onderzoek)

Summary

Rationale:

In chronic hemodialysis (HD) patients managed with thrice weekly HD, the mortality due to cardiovascular events remains high despite of all the technological improvements of this therapy in the last years. It has been shown that an increased vascular calcification is directly correlated to an increased cardiovascular mortality. In dialysis patients abnormalities in mass transport of calcium and phosphate, which are involved in formation of calciprotein particles (CPPs) could play a pathogenic role. The calcification propensity of serum, measured by a novel T_{50} test, measures the transformation time from primary to secondary CPPs and is highly predictive of all-cause mortality in HD patients. In a recent study it was shown that phosphate removal during dialysis strongly improved the T_{50}. However, less is known on the influences of dialysate calcium on the formation of CPP or on the role of calcium-citrate dialysate, in which citrate is a calcium chelator.

Objectives:

The first objective of this study is to evaluate the effect of standard HD with different dialysate calcium concentrations as well as HD combined with citrate-acid dialysate on the clearance of CPPs and second the effect of these different solutions on cardiovascular parameters.

Study design:

Chronic hemodynamically stable HD patients, with a dialysis vintage of at least 3 months, will be prospectively followed up in a cross-over randomized study.

Intervention:

In the study 22 HD patients will be treated in a randomized order with either a dialysate calcium (DCa) of 1.25 mmol/l (DCa 1.25), a DCa of 1.50 mmol/l (DCa 1.50), or citrate-acid dialysate (containing 1.5 mmol/l calcium) for 3 treatments (1 week) each.

Main study parameters/endpoints:

- Calcification propensity assessed by the transition time T_{50} from primary to secondary CPP by time-resolved nephelometry (T_{50}).
- Pulse wave velocity (PWV) measured with a SphygmoCor pulse wave velocity meter.

- Heart rate variability (HRV) measured with a Taskforce monitor.
- Total mass balances of calcium and phosphate by direct dialysis quantification.

Nature and extent of the burden and risks associated with participation, benefit and group relatedness:

In this study, only non-invasive techniques which pose a minimal burden to the patient will be used. Blood sampling, HRV-, and the PWV-measurements will be combined with the regular blood takings when patients are present in their dialysis unit. The different dialysate calcium concentrations as well as citric-acid dialysate are registered products and used in routine practice.

1. Introduction and rationale

Despite ongoing technical improvements in both dialysis and overall patient care, the annual mortality of patients with end-stage renal disease (ESRD) managed with thrice-weekly hemodialysis (HD), with as main cause of death cardiovascular disease, remains high. An important risk factor for cardiovascular mortality are vascular calcifications [1,2]. Despite all these technical improvements and the introduction of several new phosphate binding drugs and calcimimetics in the last 10 to 15 years, vascular calcification in dialysis patients remains high.

Calcification and cardiovascular morbidity and mortality

It is known that cardiovascular calcifications are common in patients with ESRD [1]. Aortic calcification can lead to an increase in vascular stiffness, which increases the systolic burden to the heart and as such a risk factor for left ventricular hypertrophy but also an independent predictor of outcome in dialysis patients [2]. Numerous studies have been performed on the pathophysiological mechanisms behind this calcifications. These data support that elevated levels of phosphorus are crucial in its pathogenesis by transforming vascular smooth muscle cells into osteoblast-like cells, which can produce a matrix of bone collagen and non-collagenous proteins [2-4].

Extracellular phosphate exerts its cytotoxic effects by forming insoluble nanoparticles with calcium and fetuin-A; these nanoparticles are referred to as calciprotein particles (CPPs) [5,6].

CPPs are highly bioactive ligands that can induce various cellular responses, including the osteogenic transformation of smooth muscle cells and cell death of vascular endothelial cells and renal tubular epithelial cells [3,4]. CCPs are detected in the serum of animal models of kidney disease and in patients with chronic kidney disease (CKD) and might be associated with a (mal)adaptation of the endocrine axes mediated by fibroblast growth factor-23 (FGF-23) and Klotho, the co-enzyme of FGF-23, that regulate phosphate homeostasis and ageing [3]. These observations raise the possibility that CPP contribute to the pathogenesis of CKD and be a reflection of the calcification propensity of uremic serum [5,6].

The formation of CPPs is a two-step process, first calcium, phosphate and fetuin A bind together to form an amorphous colloidial calcium-phosphate nanoparticle called primary CPP. Secondly they transform into topologically stable elongate spindle-shaped structures containing proteins and hydroxyl-apatite,

secondary CPP. The transition time (T_{50}) between primary and secondary CPP is thought to reflect the intrinsic inhibitory capacity of the serum to prevent calcium and phosphate from precipitating [6]
.

Another important factor in the pathogenesis of vascular calcification is the serum calcium level, though its effect is less consistent and more debated as compared to hyperphosphatemia [7]. An important factor affecting the change in serum calcium during dialysis, is the dialysate calcium (DCa) concentration. At present, DCa concentrations of 1.25 mmol/l and 1.5 mmol/l are used in clinical practice, evidence which solution is preferable is however lacking. Lower DCa (1.25 mmol/l) concentrations might influence the blood pressure response during dialysis and increase the risk of intra-dialytic hypotension, and may lead to worsening of hyperparathyroidism [8]. Moreover, a lower DCa might influence heart rate variability during dialysis and lead to a prolongation of the QT interval [9], although an adverse effect on cardiovascular outcome was only observed for Dca levels below 1.25 mmol/l (i.e. 1.0 mmol/l), which are not used in routine clinical practice [10]. A DCa of 1.5 mmol/L may be associated with improved hemodynamic tolerance during dialysis, but also increased risk of vascular calcification [11,12], although the later has not yet been proven “in vivo”. Higher DCa concentrations may also lead to an acute increase in vascular stiffness during dialysis due to influx of ionized calcium into the vascular wall, although this has only been studied for a DCa of 1.75 mmol/L, which is not commonly used in clinical practice [8].

The optimal DCa concentration in dialysis patients has led to significant debate in the community. Even in international well established guidelines as K/DOQI (DCa of 1.25 mmol/L) and KDIGO (DCa between 1.25 mmol/L and 1.50 mmol/L) there is a substantial debate in what the optimal DCa should be [13,14].This debate is mainly driven by theoretical premises, whereas data on clinical endpoints, but also on surrogate markers of calcification, such as CPP, are lacking.

The first aim of the study is to assess the calcification propensity of routinely used DCa concentrations, by assessing its effect on T_{50}.

Citrate acid-based dialysate

Citric acid-based dialysate was initially used as anticoagulant in intensive care patients on continuous renal replacement therapy or HD [15]. Citric acid-based dialysate is obtained by substituting citric acid for acetic acid in the acid concentrate. Citric acid reacts with bicarbonate to give citrate ion. The citrate concentration in the dialysate is 0.8 mmol/L, which is about one fourth (25%) of the concentration

necessary to obtain regional anticoagulation by infusion of citrate upstream of the dialyzer. Citrate chelates ionized calcium in the dialysate. This provokes a decrease in ionized calcium concentration in dialysate, inducing diffusion of ionized calcium from blood to dialysate. These mechanisms result in a decrease in ionized calcium in the blood circuit, producing a local anticoagulant effect. A study in 2000 showed that measured ionized calcium in patients decreased only slightly when citric acid-based dialysate was used and no infusion of calcium ion was required [16]. In a more recent study citric acidbased dialysate improved as compared with acid-based dialysate in conventional HD the removal of phosphate and 2-microglobulin [17,18].

Next to this, citrate-acid buffer is increasingly used as an anticoagulant during HD. Also at our dialysis department and intensive care unit, citric acid-based dialysate has been used for various years during chronic intermittent HD and continuous veno-venous hemodiafiltration (CVVHDF) as part of routine (HD) and acute (CVVHDF) treatment without any problems. The use of citric acid-based dialysate has also been proven safe in several HDF studies [19-21].

Summarizing on theoretical and pathophysiological grounds citric acid-based dialysate may act as calcium chelating and further improves the calcification propensity, as assessed by T_{50}. The second aim of the study is to assess the effect of citric-acid based dialysate on calcification propensity, assessed by T_{50}.

2. Objectives

The aim of this prospective randomized controlled trail is to study the effect of standard HD with different dialysate calcium concentrations as well as HD combined with citrate-acid dialysate on calcification propensity assessed by CPPs . The secondary aim is to assess the effect of these different solutions on cardiovascular parameters in detail .

3. Study design

Twenty-two prevalent conventional high-flux HD (CHD) patients will undergo, in a random prospective design, 1 week standard high-flux HD with DCa1.50 (treatment A) followed by either citrate aciddialysate HD (treatment B) or high-flux HD with DCa1.25 (treatment C) for 1 week, followed by a washout period of 1 week on standard high-flux HD with DCa1.50 (treatment A), followed by either citrate acid-dialysate HD (treatment B) or high-flux HD with DCa1.25 (treatment C) for 1 week. Due to patient monitoring during the weeks when different concentrated dialysate fluids are used, it is not possible to blind participating patients or the researchers in this study.

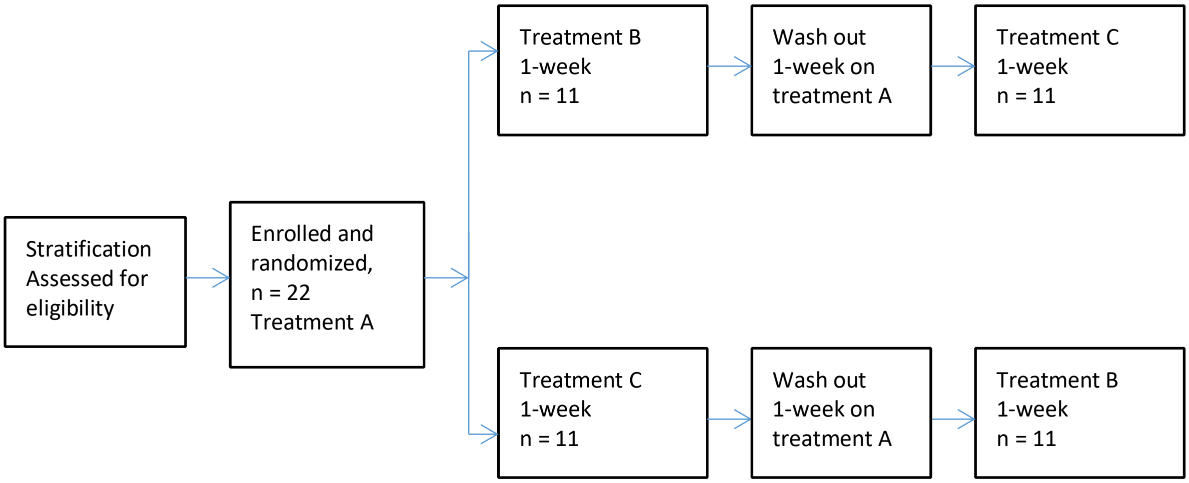

Figure 1: study design, prospective randomized cross-over trail.

4. Study population

4.1 Population

For this study 22 stable chronic HD patients will be included from the dialysis unit of the Maastricht University Medical Center and the Catharina Hospital in Eindhoven. Due to the cross over design of this study patients will be their own control and a control group will not be necessary.

4.2 Inclusion criteria

- Prevalent HD patients with a dialysis vintage of at least 3 months.
- Hemodynamically stable on dialysis.
- AV-fistula enabling double-needle vascular access or tunneled central venous dialysis catheter with a blood flow rate of at least 300 ml/min Age above 18 years of age.
- Informed consent.

4.3 Exclusion criteria

- Withdrawal of consent
- Acute intercurrent illness (infection, malignancy, cardiovascular event, uncontrolled diabetes)
- Long QT syndrome (QTc >470 ms)
- Frequent intra-dialytic hypotension (>10% of treatments)

4.4 Sample size calculation

The power analysis is based on a hypothesized 20% difference in T_{50} between the different treatment modalities. In the population of Smith et al. [22], T_{50} in the lowest tertile was 277 ±44 min. In a previous pilot study (MEC-U 2014-31), the T_{50} in a hemodialysis and Hemodiafiltration of 30 and 34 patients were 236 ± 60 and 147 ± 58 min respectively. Taking these different populations into account a mean of 250 min and a SD of 55 min were used as the basis of the calculations. With a power of 80% and an alpha level of 0.05, 19 patients would be needed to show significant differences between the different HD treatments in a pairwise analysis. To allow for multiple comparisons, and a lost to follow up percentage of 10%, 22 patients will be asked to participate.

5. Non-Investigational Product

5.1 Name and description of non-investigational product(s)

To investigate the potential modifying effect of CPP T50 by dialysate calcium concentration (DCa), in this study we will use the standard 1.50 mmol/L Ca dialysate as a reference group. In the “intervention” weeks we will use the standard 1.25 mmol/L dialysis fluid of Fresenius Medical care (AC-F 213/313, depending on the patients potassium profile), in the weeks of the low dialysate fluid. In the week of de citrate dialysis fluid we will use the Fresenius Medical Care Smartbag 211 or 311, again adjusted to the patients own potassium level.

For detailed information I would like to refer to the Summary of Product Characteristics (SPC) added to this protocol as appendix I (AC-F) and Appendix II (smartbag).

5.2 Dosages, dosage modification and method of administration

In this study the studied dialysate fluids will be administered according to the normal protocols. No alterations in volume or method of administration are present in this current study.

6. Methods

6.1 Study parameters/endpoints

6.1.1 Primary endpoint.

- T_{50} measured by time resolved nephelometry in HD with citrate-acid dialysate.

Secondary endpoints:.

- Acute changes in arterial stiffness.
- Changes in HRV, measured by Task Force measurements, due to changes in ionized calcium with different DCa concentrations.
- Relation between

6.1.2 Safety endpoints:

- prolonged QTc interval (QTc>470ms)
- the occurrence of interdialytic hypotension during two consecutive dialysis treatments
- severe unresponsive hyperparathyroidism

6.2 Randomisation

In the study 22 HD patients will be treated in a randomized order with either a dialysate calcium (DCa) of 1.25 mmol/l (DCa 1.25), a DCa of 1.50 mmol/l (DCa 1.50), or citrate-acid dialysate (containing 1.5 mmol/l calcium) for 3 treatments (1 week) each. Patients will be randomized en-block after inclusion of 5 patients, using the online programme of randomization.org.

6.3 Study procedures

Clinical data and patients characteristics:

Clinical data and patients characteristics will be collected from the medical patient files and from patients while they are in the dialysis unit for their treatment. Dialysis parameters as blood pressure, pulse rate and dry body weight, as well as base line characteristics as age, race, gender, primary kidney disease, smoking, medication use and history are included.

Laboratory measurements

Blood samples will be taken every dialysis before and after dialysis while patients are in the dialysis unit and are connected to the dialysis machine, so no extra puncture will be necessary.

Blood sample analysis and Measurements

Serum samples will be taken from the inlet bloodlines immediately before the onset and at the end of the dialysis session. The samples will be analysed for magnesium, calcium, ionized calcium, albumin, phosphate, bicarbonate, hematocrit, and the nephelometric assessment of transition from primary to secondary CPP. For this last analysis, the samples will be centrifuged at room temperature (20 degrees Celcius) for 15 minutes, within 240 after collection and freezed immediately with a freezing temperature of -70 ^{0}C,. Than the samples are transported to the University Hospital of Bern where the samples are analysed within 72 hours after collection. The serum fetuin A concentration will be measured by nephelometry, a test first establisched by the Jahnen-Dechent group [5]. CPP-T50 will be determined as described by the method by Pasch et al. [6]. Samples will be supersaturated by adding Ca (10 mM) and P (6mM) to initiate the formation of primary CPP. The time of spontaneous transformation to secondary CPPs will be measured by a nephelostar nephelometer. Also an additional 5ml tube will be collected and stored for possible additional analyses for this current study.

Dialysate Sampling and Measurements

A mixture of spent dialysate and ultrafiltrate will continuously be collected in a fractionated fashion in a bag. At the end of the treatment and after thorough mixing, a 10 mL sample will be drawn from the collection bag in order to quantify solute concentration. All samples will stored at - 80˚C until analysis.

Monitoring during dialysis:

ECG analysis

An ECG will be performed before and after the first treatment session with citrate acid-based HD, followed by the same procedure 1 week later. Patients with long QT syndrome or prolonged QT interval after HD with citrate acid based-dialysate will be excluded from the study.

Task Force Monitor Measurements

Impedance cardiography measures intra thoracic fluid shifts during a cardiac cycle. Task Force Monitor will be used as described elsewhere[23]. Three electrodes will be placed on the patient, one in the neck close to the glottis and two below the thorax close to the xiphoidea. With the task force monitor we are able to measure cardiac output, stroke volume and heart rate by a non-invasive method during the complete dialysis session. This allows us to monitor patients adequately when the lower calcium

concentration dialysate and the citrate dialysate fluids are used. Any ECG changes and hemodynamic changes will be registered directly and can lead to timely intervention. The Task Force Monitor will be used during the one dialysis session in week 1, 2 and 4, in order to combine study measurements wit safety measurements (continuous heart rate monitoring is available in the Task Force Monitoring). Patient will be monitored during the next two dialysis sessions with a the conventional ECG, heart rate and blood pressure monitor that is also used to monitor critically ill patients at the ICU and emergency room wards.

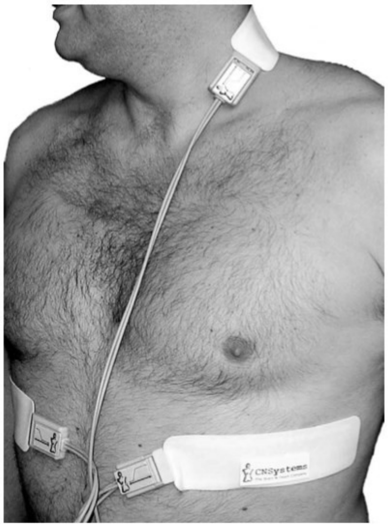

Figure 2: placement of the electrodes for Task force measurements.

Pulse wave velocity (PWV) measurements

The pulse wave velocity measurement will be done once at the start and once at the end of the study and also when the patients are in the dialysis unit for their treatment. The PWV measurements will be performed with a SphygmoCor device. The peripheral pulse wave is measured at the a.radialis by applanation tonometry and recorded on a personal computer. The quality of the recording can be appreciated using the provided operator index. The pulse wave than has to be calibrated by externally determined blood pressure values. The computer software of the SphygmoCor calculates the aortic pulse wave using a transfer function. This transformation provides the first parameter under investigation, the aortic systolic blood pressure and the aortic pulse pressure. Now a characteristic point of the pressure curve, the inflection point, is identified within the time domain, indicating the arrival of the reflected wave in the ascending aorta. The blood pressure at this point of time is called inflection pressure. The differences between aortic systolic blood pressure and inflection pressure is called augmentation pressure.

Dialysate composition

Citrate acid-based HD: sodium 138 mmol/l, potassium 2-4 mmol/l, magnesium 0.5 mmol/l, calcium 1.5 mmol/l, glucose 1 g/l, acetate 0 mmol/l, citrate 1mmol/l, bicarbonate 32 mmol/l.

Standard high-flux HD: sodium 138 mmol/l, potassium 2-3 mmol/l, magnesium 0.5 mmol/l, calcium 1.25 or 1.5 mmol/l, acetate 3 mmol/l, bicarbonate 32 mmol/l.

All measurements have been chosen because they can be performed non-invasively, without significant burden for the patient. Total time investment to undergo all the tests is estimated to be minimal.

6.4 Withdrawal of individual subjects

Subjects can leave the study at any time for any reason if they wish to do so without any consequences. The investigators can decide to withdraw a subject from the study for urgent medical reasons.

6.4.1. Specific criteria for withdrawal (if applicable)

As this is a non-invasive study, no specific withdrawal criteria are formulated, except for withdrawal of consent.

6.5 Replacement of individual subjects after withdrawal

Patients who withdraw from the study will not be replaced, as this has already been taken into account in the simple size calculation.

6.6 Follow-up of subjects withdrawn from treatment

Patients who withdraw from the study will be not be followed.

6.7 Premature termination of the study

In accordance with the research contract this study may be terminated directly:

- if the judgement of the competent Medical Ethics Committee has assessed this study is irrevocably revoked.
- If a reasonable case can be made for terminating this study in the interests of the health of the research subjects.

- If it transpires that continuation of this study cannot serve any scientific purpose, and this is confirmed by the Medical Ethics Committee that has issued a positive decision on the study.
- If one of the parties had been declared insolvent or a bankruptcy/winding-up petition has been filed in respect of one of the parties, or if one of the parties is dissolved as a legal entity.
- If the principal investigator is no longer capable of performing his task, and no replacement acceptable to both parties can be found in 45 days.
- If one of the parties fails to comply with the obligations arising from the project agreement and, provided compliance is not permanently impossible, this compliance has not taken place within thirty days of the defaulting party receiving a written request to comply, unless failure to comply is not in reasonable proportion to the premature termination of the study.
- If circumstances beyond the control of a party or the principal investigator make it unreasonable to require the study's continuation.

7. Safety reporting

7.1 Temporary halt for reasons of subject safety

In accordance to section 10, subsection 4, of the WMO, the sponsor will suspend the study if there is sufficient ground that continuation of the study will jeopardise subject health or safety. The sponsor will notify the accredited METC without undue delay of a temporary halt including the reason for such an action. The study will be suspended pending a further positive decision by the accredited METC. The investigator will take care that all subjects are kept informed.

7.2 AEs, SAEs and SUSARs

7.2.1. Adverse events (AEs)

Adverse events (AE) are defined as any undesirable experience occurring to a subject during a study, whether or not considered related to the experimental intervention. All adverse events reported spontaneously, with the exception of expected adverse events as bruises, by the subject observed by the investigator or his staff will be recorded.

7.2.2. Serious adverse events (SAEs)

A serious adverse event is any untoward medical occurrence or effect that

- results in death;

- is life threatening (at the time of the event);
- requires hospitalisation or prolongation of existing inpatients' hospitalisation;
- results in persistent or significant disability or incapacity;
- any other important medical event that did not result in any of the outcomes listed above due to medical or surgical intervention but could have been based upon appropriate judgement by the investigator.

An elective hospital admission will not be considered as a serious adverse event.

The investigator will report all SAEs to the sponsor without undue delay after obstaning knowledge of the events.

The sponsor will report the SAEs through the web portal Toetsingonline to the accredited METC that approved the protocol, within 7 days of first knowledge for SAEs that result in death or are life threatening followed by a period of maximum of 8 days to complete the initial preliminary report. All other SAEs will be reported within a period of maximum 15 days after the sponsor has first knowledge of the serious adverse events.

A serious adverse event which requires hospitalisation or prolongation of existing inpatients' hospitalisation and is **not study-related** will be recorded by the researcher(s) and reported to the accredited METC in an overview attached to the yearly progress report.

7.2.3 Suspected unexpected serious adverse reactions (SUSAR)

Not applicable

7.4 Follow-up of adverse events

Adverse events according to the study are considered very unlikely, due to the fact that only noninvasive methods are used. All clinical events which happen in this group of patients will be followed and treated according to standard procedures.

7.5 Data Safety Monitoring Board (DSMB)

No DSMB is installed, as this is an observational study with non-invasive methods.

8. Statistical analysis

Data will be presented as mean ± standard deviation. Repeated measurements ANOVA will be used to compare the outcomes of the 3 different dialysis modalities. The tests will be considered significant if pvalues are below 0.05.

9. Ethical Considerations

9.1 Recruitment and consent

All patients will receive an information leaflet by the treating nephrologist. In this leaflet, information regarding the aims and conduct of the study, the burden and risks associated with participation. If the patient is interested in participation, he or she will mention his or her interest in the dialysis unit and subsequently, they will be contacted by the investigators (the nephrologist in training and/or research nurse) who will also explain the procedure verbally in detail. Each participant will have at least 1 week time to decide whether he/she wishes to participate in the study. The consent and insurance procedures will be explained by the nephrologist-researcher and/or the research nurse. After signing the informed consent form, the study will be initiated.

9.2 Objection by minors or incapacitated subjects (if applicable)

Not applicable

9.3 Benefits and risks assessment, group relatedness

The predominant safety aspect of this protocol is the possible effect of citrate based dialysate on plasma levels of ionized calcium, as discussed in the protocol. No safety effects of this small decline have been observed. However, during the first week of treatment with citrate based OL-HDF, pre- and postdialytic plasma levels of ionized calcium will be assessed during two subsequent treatments, followed by an analysis once weekly afterwards. Patients with long QT syndrome will be excluded by performing an ECG before inclusion. Moreover, the acid base status of the patient will be assessed in a similar way by assessing total CO_2 levels in a similar way as described for plasma ionized calcium levels. In addition an ECG will be performed before and after the first treatment session with citric acid-based OL-HDF, followed by the same procedure 3 weeks later.

We expect that the study will have no direct benefits for the participating patients. This will also be mentioned in the patient information and on the informed consent form.

This study can provide valuable new information on the prevention of arterial calcification and thereby possibly reducing cardiovascular morbidity and mortality in the foreseeable future. Therefore the investigators believe that the risk-benefit ratio of this study is in balance.

9.4 Compensation for injury

The sponsor/investigator has a liability insurance which is in accordance with article 7 of the WMO.

The sponsor (also) has an insurance which is in accordance with the legal requirements in the Netherlands (Article 7 WMO). This insurance provides cover for damage to research subjects through injury or death caused by the study. The insurance applies to the damage that becomes apparent during the study or within 4 years after the end of the study.

9.5 Incentives (if applicable)

No special incentives will be given to the participants. If the patient wishes so, the results of the study are discussed with an explained to the patient.

10. Administrative aspects and publication

10.1 Regulation statement

The study will be conducted according to the principles of the Declaration of Helsinki (Brasil 2013) and in accordance with the Medical Research Involving Human Subjects Act (WMO) and other guidelines, regulations and acts.

10.2 Handling and storage of data, documents and body materials

The personal data will be coded via study numbers; only the researches have the key to these codes. Furthermore, only researchers have access to the original sources. The data will be stored for a 15 years including the time for further research.

The blood samples drawn during this study will be analysed directly in the laboratory of the MUMC+ or the Catharina Hospital Eindhoven, depending on the dialysis localisation of the patient. The tubes will be numbered with patients study numbers that will not contain any identifiable information. Only the primary investigator will have the key to these codes. A small amount of these samples will be send to Bern for the additional fetuin A and CPP analysis. These samples are also numbered with patient study numbers of which only the primary investigator has the key, this key remains in The Netherlands, the

analyses performed in Bern will be done with samples completely anonimyzed. Any residual material of the samples send to Bern, will be destroyed directly after the CPP analysis and fetuin A measurement.

After the analyses any remaining patient blood samples will be stored in the refrigerator to the end of the study. At the end of the study the samples will be stored for possible further research in the future, in the line of this current protocol, in the Catharina Hospital Eindhoven and the MUMC+.

During the study not only the researchers but also members of the Inspectie van de Gezondheidszorg, safety monitors and members of the Medical Ethical Committee have access to the data of the study.

10.3 Amendments

All amendments will be notified to the METC that gave favourable opinion.

10.4 Annual progress report

The sponsor/investigator will submit a summary of the progress of the trial to the accredited METC once a year. Information will be provided on the date of inclusion of the first subject, numbers of subjects included and numbers of subjects that have completed the trail, serious adverse events/serious adverse reactions, other problems and amendments.

10.5 End of study report

The investigator will notify the accredited METC of the end of the study within a period of 8 weeks. The end of the study is defined as the last patient’s dialysis.

In case the study is ended prematurely, the investigator will notify the accredited METC, including the reasons for the premature termination.

Within one year after the end of the study, the investigator/sponsor will submit a final study report with the results of the study, including any publications/abstracts of the study, to the accredited METC, including the reasons for the premature termination.

10.6 Public disclosure and publication policy

The publication policy is based on the Central Committee on Research Involving Human Subjects statement (CCMO) of November 2011. All results will be made known, and will be offered for publication in peer-reviewed journals.

References

1. London GM, Guerin AP, Marchais SJ, Metivier F, Pannier B, Adda H. Arterial media calcification in end-stage renal disease: impact on all-cause and cardiovascular mortality. *Nephrology, dialysis, transplantation : official publication of the European Dialysis and Transplant Association - European Renal Association.* Sep 2003;18(9):1731-1740.

2. Moe SM, Chen NX. Pathophysiology of vascular calcification in chronic kidney disease. *Circulation research.* Sep 17 2004;95(6):560-567.

3. Kuro-o M. Klotho, phosphate and FGF-23 in ageing and disturbed mineral metabolism. *Nature reviews. Nephrology.* Nov 2013;9(11):650-660.

4. Smith ER, Ford ML, Tomlinson LA, Rajkumar C, McMahon LP, Holt SG. Phosphorylated fetuin-Acontaining calciprotein particles are associated with aortic stiffness and a procalcific milieu in patients with pre-dialysis CKD. *Nephrology, dialysis, transplantation : official publication of the European Dialysis and Transplant Association - European Renal Association.* May 2012;27(5):1957-1966.

5. Smith ER, Ford ML, Tomlinson LA, et al. Serum calcification propensity predicts all-cause mortality in predialysis CKD. *Journal of the American Society of Nephrology : JASN.* Feb 2014;25(2):339-348.

6. Pasch A, Farese S, Graber S, et al. Nanoparticle-based test measures overall propensity for calcification in serum. *Journal of the American Society of Nephrology : JASN.* Oct 2012;23(10):1744-1752.

7. Khoshniat S, Bourgine A, Julien M, et al. Phosphate-dependent stimulation of MGP and OPN expression in osteoblasts via the ERK1/2 pathway is modulated by calcium. *Bone.* Apr 1 2011;48(4):894-902.

8. Kyriazis J, Katsipi I, Stylianou K, Jenakis N, Karida A, Daphnis E. Arterial stiffness alterations during hemodialysis: the role of dialysate calcium. *Nephron. Clinical practice.* 2007;106(1):c34-42.

9. Nappi SE, Virtanen VK, Saha HH, Mustonen JT, Pasternack AI. QTc dispersion increases during hemodialysis with low-calcium dialysate. *Kidney international.* May 2000;57(5):2117-2122.

10. Miller JE, Kovesdy CP, Norris KC, et al. Association of cumulatively low or high serum calcium levels with mortality in long-term hemodialysis patients. *American journal of nephrology.* 2010;32(5):403-413.

11. Gotch F, Kotanko P, Levin N. How can we improve the solute and fluid transport prescriptions in hemodialysis to improve patient outcomes? *Blood purification.* 2013;35(1-3):93-105.

12. Yamada K, Fujimoto S, Nishiura R, et al. Risk factors of the progression of abdominal aortic calcification in patients on chronic haemodialysis. *Nephrology, dialysis, transplantation : official publication of the European Dialysis and Transplant Association - European Renal Association.* Jul 2007;22(7):2032-2037.

13. Gotch FA, Kotanko P, Thijssen S, Levin NW. The KDIGO guideline for dialysate calcium will result in an increased incidence of calcium accumulation in hemodialysis patients. *Kidney international.* Aug 2010;78(4):343-350.

14. Moe SM, Drueke TB. The KDIGO guideline on dialysate calcium and patient outcomes: need for hard evidence. *Kidney international.* Feb 2011;79(4):478; author reply 478-479.

15. Schmitz M, Taskaya G, Plum J, et al. Continuous venovenous haemofiltration using a citrate buffered substitution fluid. *Anaesthesia and intensive care.* Aug 2007;35(4):529-535.

16. Ahmad S, Callan R, Cole JJ, Blagg CR. Dialysate made from dry chemicals using citric acid increases dialysis dose. *American journal of kidney diseases : the official journal of the National Kidney Foundation.* Mar 2000;35(3):493-499.

17. Kossmann RJ, Gonzales A, Callan R, Ahmad S. Increased efficiency of hemodialysis with citrate dialysate: a prospective controlled study. *Clinical journal of the American Society of Nephrology : CJASN.* Sep 2009;4(9):1459-1464.

18. Gabutti L, Lucchini B, Marone C, Alberio L, Burnier M. Citrate- vs. acetate-based dialysate in bicarbonate haemodialysis: consequences on haemodynamics, coagulation, acid-base status, and electrolytes. *BMC nephrology.* 2009;10:7.

19. Aniort J, Petitclerc T, Creput C. Safe use of citric acid-based dialysate and heparin removal in postdilution online hemodiafiltration. *Blood purification.* 2012;34(3-4):336-343.

20. Grundstrom G, Christensson A, Alquist M, Nilsson LG, Segelmark M. Replacement of acetate with citrate in dialysis fluid: a randomized clinical trial of short term safety and fluid biocompatibility. *BMC nephrology.* 2013;14:216.

21. Panichi V, Fiaccadori E, Rosati A, et al. Post-dilution on line haemodiafiltration with citrate dialysate: first clinical experience in chronic dialysis patients. *TheScientificWorldJournal.* 2013;2013:703612.

22. Smith ER, Hanssen E, McMahon LP, Holt SG. Fetuin-A-containing calciprotein particles reduce mineral stress in the macrophage. *PloS one.* 2013;8(4):e60904.

23. Fortin J, Habenbacher W, Heller A, et al. Non-invasive beat-to-beat cardiac output monitoring by an improved method of transthoracic bioimpedance measurement. *Computers in biology and medicine.* Nov 2006;36(11):1185-1203.
